# Favipiravir Effects on the Control of Clinical Symptoms of Hospitalized COVID-19 Cases: An Experience with Iranian Formulated Dosage Form

**DOI:** 10.22037/ijpr.2021.115510.15401

**Published:** 2021

**Authors:** Payam Tabarsi, Hossein Vahidi, Ali Saffaei, Seyed Mohammad Reza Hashemian, Hamidreza Jammati, Bahram Daraei, Arash Mahboubi, Farzad Kobarfard, Majid Marjani, Afshin Moniri, Zahra Abtahian, Atefeh Abedini, Alireza Eslaminejad, Jalal Heshmatnia, Maryam Sadat Mirenayat, Atefeh Fakharian, Sharareh Seifi, Mohsen Sadeghi, Alireza Dastan, Sara Haseli, Seyed Alireza Nadji, Raha Eskandari, Sahar Yousefian, Mohammad Varahram, Alireza Zali, Ali Akbar Velayati, Farzaneh Dastan

**Affiliations:** a *Clinical Tuberculosis and Epidemiology Research Center, National Research Institute of Tuberculosis and Lung Diseases (NRITLD), Masih Daneshvari Hospital, Shahid Beheshti University of Medical Sciences, Tehran, Iran. *; b *Department of Pharmaceutical Biotechnology, School of Pharmacy, Shahid Beheshti University of Medical Sciences, Tehran, Iran. *; c *Student Research Committee, Department of Clinical Pharmacy, School of Pharmacy, Shahid Beheshti University of Medical Sciences, Tehran, Iran. *; d *Chronic Respiratory Diseases Research Center (CRDRC), National Research Institute of Tuberculosis and Lung Diseases (NRITLD), Shahid Beheshti University of Medical Sciences, Tehran, Iran. *; e *Department of Toxico/Pharmacology, School of Pharmacy, Shahid Beheshti University of Medical Sciences, Tehran, Iran.*; f *Department of Pharmaceutics and Nanotechnology, School of Pharmacy, Shahid Beheshti University of Medical Sciences, Tehran, Iran. *; g *Department of Medicinal Chemistry, School of Pharmacy, Shahid Beheshti University of Medical Sciences, Tehran, Iran. *; h *Ernest and Julio Gallo Management Program, School of Engineering, University of California, Merced, CA, United States. *; i *Virology Research Center, National Research Institute of Tuberculosis and Lung Diseases (NRITLD), Masih Daneshvari Hospital, Shahid Beheshti University of Medical Sciences, Tehran, Iran. *; j *Functional Neurosurgery Research Center, Shohada Tajrish Comprehensive Neurosurgical Center of Excellence, Shahid Beheshti University of Medical Sciences, Tehran, Iran. *; k *Tracheal Diseases Research Center, National Research Institute of Tuberculosis and Lung Diseases (NRITLD), Shahid Beheshti University of Medical Sciences, Tehran, Iran. *; l *Department of Clinical Pharmacy, School of Pharmacy, Shahid Beheshti University of Medical Sciences, Tehran, Iran.*

**Keywords:** COVID-19, SARS-CoV-2, Favipiravir, Lopinavir-ritonavir, Antiviral

## Abstract

Coronavirus disease -19 (COVID-19) pandemic, caused by SARS-CoV-2, has gradually spread worldwide, becoming a major public health event. This situation requires designing a novel antiviral agent against the SARS-CoV-2; however, this is time-consuming and the use of repurposed medicines may be promising. One such medicine is favipiravir, primarily introduced as an anti-influenza agent in east world. The aim of this study was to evaluate the efficacy and safety of favipiravir in comparison with lopinavir-ritonavir in SARS-CoV-2 infection. In this randomized clinical trial, 62 patients were recruited. These patients had bilateral pulmonary infiltration with peripheral oxygen saturation lower than 93%. The median time from symptoms onset to intervention initiation was seven days. Favipiravir was not available in the Iranian pharmaceutical market, and it was decided to formulate it at the research laboratory of School of Pharmacy, Shahid Beheshti University of Medical Sciences, Tehran, Iran. The patients received favipiravir tablet at a dose of 1600 mg orally twice a day for day one and then 600 mg orally twice a day for days 2 to 6. In the second group, the patients received lopinavir-ritonavir combination tablet at a dose of 200/50 mg twice a day for seven days. Fever, cough, and dyspnea were improved significantly in favipiravir group in comparison with lopinavir-ritonavir group on days four and five. Mortality rate and ICU stay in both groups were similar, and there was no significant difference in this regard (*P = *0.463 and *P = *0.286, respectively). Chest X-ray improvement also was not significantly different between the two groups. Adverse drug reactions occurred in both groups, and impaired liver enzymes were the most frequent adverse effect. In conclusion, early administration of oral favipiravir may reduce the duration of clinical signs and symptoms in patients with COVID-19 and hospitalization period. The mortality rate also should be investigated in future clinical trials.

## Introduction

Novel coronavirus (COVID-19) pneumonia as a pandemic disease spreads across 213 countries. Up to 20 April 2021, 142,613,055 cases have been diagnosed with COVID-19, and among them, 3,041,038 cases have died. In the absence of specific treatment for COVID-19, there is an urgent need to find alternative treatments to manage this pandemic (1). To date, no specific therapy for COVID-19 pneumonia currently exists, and patients’ treatment remains a great challenge. Several agents, such as chloroquine, hydroxychloroquine, lopinavir/ritonavir, remdesivir, intravenous immunoglobulin, interferons, tocilizumab, and immune modulatory agents, are currently under investigation for the treatment of COVID-19 (2-4). 

Among antiviral agents, favipiravir (T-705) has broad-spectrum activity against Ribonucleic acid (RNA) viruses, including influenza virus, rhinovirus, and respiratory syncytial virus. Studies on seasonal influenza that have been conducted in Japan and United States showed promising results on re-emerging influenza viruses (5). In animal models, favipiravir has been reported to be effective for prophylaxis and treating Ebola virus infection. In addition, favipiravir was successfully used for post-exposure prophylaxis and treatment in patients with Ebola virus infection (6).

An early study showed the efficacy of favipiravir against SARS-CoV-2 in china (7). However, there are insufficient pieces of evidence in this regard. At the beginning of the pandemic, the favipiravir sources were limited in the Iranian pharmaceutical market, and it was necessary to formulate this drug by internal companies. Here, we aimed to evaluate the efficacy and safety of Iranian formulated favipiravir in comparison with lopinavir-ritonavir in COVID-19 infection. 

## Experimental

*Patients and setting*

This study was a randomized prospective clinical trial done at Dr. Masih Daneshvari Hospital, a university-affiliated and selected referral center for COVID-19 patients, Tehran, Iran. The study was conducted according to the guidelines of the Declaration of Helsinki, principles of good clinical practice, and Consolidated Standards of Reporting Trials guidelines (CONSORT). The ethics committee of Shahid Beheshti University of Medical Sciences approved the protocol of the study (IR.SBMU.RETECH.REC.1399.011). The trial protocol was also registered in the Iranian Clinical Trial Center (IRCT20151227025726N14). Patient enrollment was done between 4 April 2020 and 7 May 2020. 

*Eligibility Criteria*

In this study, patients who were admitted during the mentioned period were assessed for eligibility. The inclusion criteria were as follows: 1) patients who were aged more than 18 years old; 2) Patients with laboratory-confirmed COVID-19 using Reverse Transcription-Polymerase Chain Reaction (RT-PCR) ; 3) patients with peripheral oxygen saturation less than 93%; 4) patients with persistent fever (more than 72 h before admission); and 5) patients with bilateral pulmonary infiltration. The exclusion criteria were 1) patients with impaired function of kidney and liver (defined as estimated glomerular filtration rate less than 30 mL/kg/h and child-pugh class C); 2) pregnant patients those intended to get pregnant within six months post-trial or breastfeeding patients; 3) known cases with a history of severe drug allergy; 4) mild cases of COVID-19 (defined as those with only upper respiratory tract infection); 5) critical cases of COVID-19 (defined as those needed intensive care unit admission or invasive mechanical ventilation). 

*Randomization and Allocation*

The allocation was according to the block randomization method. 16 blocks, including 4 patients in each block, were generated by the Online Randomizer website (www.sealesenvelop.com/simple randomizer). In each block, two patients were assigned to the favipiravir group, and two patients were assigned to the lopinavir-ritonavir group. Before recruitment, the eligible patients who signed the informed consent form, were introduced to the pharmaceutical care ward of the hospital. The eligibility criteria were rechecked and the patients were allocated to favipiravir or lopinavir/ritonavir groups. 

*Formulation of favipiravir*

Favipiravir was not easily available in the pharmaceutical market of Iran, and only a limited storage of favipiravir (Zhejiang Hisun Pharmaceutical Co., Ltd.,) was available at the time of the study. Hence, it was decided to formulate this drug in the research laboratory of the School of Pharmacy, Shahid Beheshti University of Medical Sciences, Tehran, Iran. The active ingredient was purchased from PharmaResources (Kaiyuan) company, Shanghai, China. 

*Intervention*

All patients received supportive care, including oxygen therapy, hydration, stress ulcer prophylaxis, deep vein thrombosis prophylaxis, and an antibiotic if it was needed. We considered empiric antimicrobial therapy just based on the Infectious Diseases Society of America guideline on community-acquired and hospital-acquired pneumonia or if the patient was immunocompromised and needed antimicrobial prophylaxis. In addition, acetaminophen (antipyretics) at a dose of 500 mg orally four times a day and expectorant syrup (cough suppressants) at a dose of 5 mL twice a day administered for all cases. Dexamethasone also was administered at a dose of 8 mg intravenously for all the patients in 10 days or at a shorter time if the patient was discharged from the hospital. Hydroxychloroquine was not administered due to the drug interaction with lopinavir-ritonavir. Patients in the favipiravir group received a favipiravir tablet at a dose of 1600 mg orally twice a day for day one and then 600 mg orally twice a day for days 2 to 6. In the other group, patients received lopinavir-ritonavir (Heterd Company, India) combination tablets at a dose of 200/50 mg twice a day for seven days.

*Outcomes*

For all the patients, demographic data, including age, sex, past medical history, and baseline laboratory results, were reported. Primary outcome was defined as changes in baseline clinical symptoms, including fever, cough, and dyspnea. Secondary outcomes also were determined as the need for admission to the Intensive Care Unit (ICU), duration of ICU stay, need for treatment with anti-inflammatory agents, changes in baseline radiological status, adverse drug reactions, hospitalization, and mortality.

*Statistical analysis*

The quantitative data were described as the mean ± standard deviation or median (interquartile range). The frequency rates were described by the number of cases (proportion or percent). The survival rate was measured using the Kaplan–Meier estimator. Results were summarized and analyzed using the Statistical Package for the Social Sciences (SPSS) v.24.0 software (IBM Corp., Armonk, NY, USA). 

## Results

*Patients and baseline characteristics*

In this study, 76 patients were included primarily, and finally, 62 patients completed the study. Fourteen patients were excluded from the study due to the non-adherence issues and occurrence of side effects. Of 62 patients, 36 (58.06%) cases were male, and the rest were female. The median age of patients was 57 years (interquartile range, 44-68 years). Thirty (48.38%) patients did not have any underlying disease, and 32 patients had at least one underlying disease. The most prevalent underlying diseases were diabetes (20.96%) and hypertension (12.90%). The baseline characteristics are shown in Table 1.

**Figure 1:**
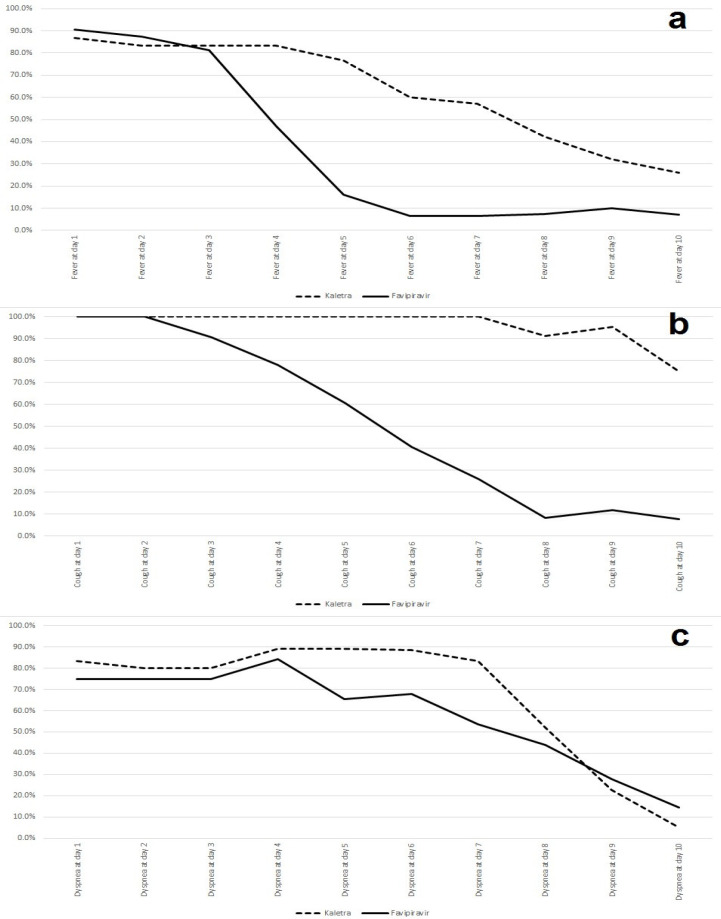
The trend of clinical symptoms in both groups. (a: fever, b: cough, and c: dyspnea).

 Changes in baseline clinical symptoms were assessed from intervention time to day 10 post-intervention. The results showed that baseline clinical symptoms status was not statistically different between the two groups. Fever was present in 26 (86.66%) patients in the lopinavir-ritonavir group and 29 (90.62%) patients in the favipiravir group (*P = *0.629). Cough was present in all patients in both groups, and dyspnea was present in 25 (83.33%) patients in the lopinavir-ritonavir group and 24 (75%) patients in the favipiravir group (*P = *0.429). Fever and dyspnea were improved significantly (*P* < 0.05) in the favipiravir group in comparison with the lopinavir-ritonavir group on day 5 and cough was resolved significantly in the favipiravir group compared to the lopinavir-ritonavir group on day 4. The trends of these symptoms are shown in Figure 1.

Five (16.66%) patients in the favipiravir group needed ICU care, while in the lopinavir-ritonavir group, eight (25%) patients needed ICU (*P = *0.286). The duration of ICU stay was 4.5 days for both favipiravir and lopinavir-ritonavir groups (IQR, 3-7 days and 2-8.5 days respectively) (*P = *0.688). Three (9.37%) patients in the favipiravir group needed anti-inflammatory agent injection, while in the lopinavir-ritonavir group, the anti-inflammatory agent was injected for five (16.66%) patients (*P = *0.467).

The adverse drug reaction occurrence results showed that two patients developed acute kidney injury, and three patients experienced liver enzyme elevation in the favipiravir group. On the other hand, acute kidney injury occurred in three patients in the lopinavir-ritonavir group, while liver enzyme elevation was seen in five patients (*P = *0.225). All the above reactions led to drug discontinuation.

**Figure 2:**
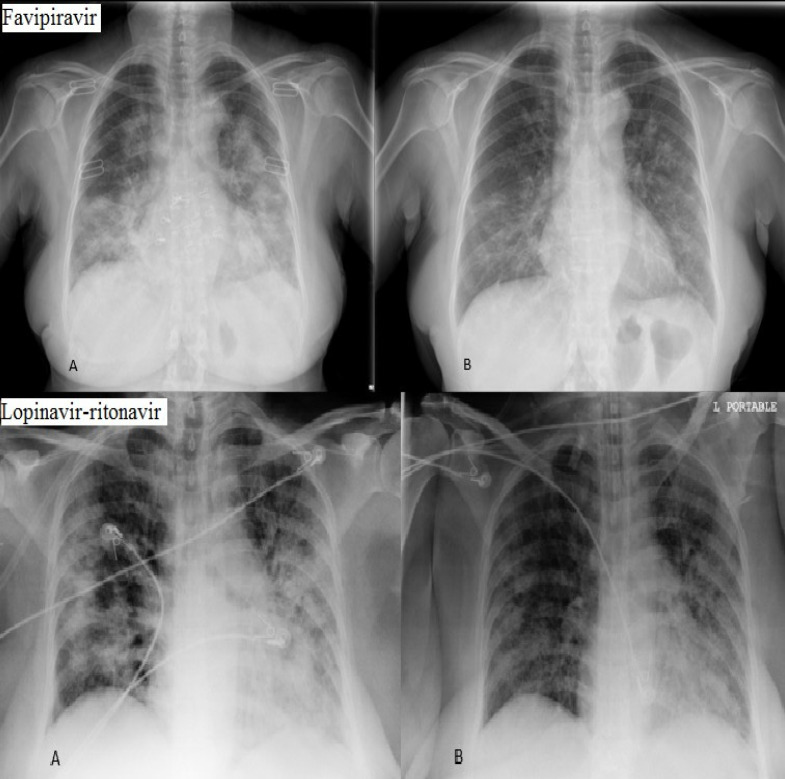
Chest X-ray improvement in two cases. (A: baseline, B: day 10)

Chest X-ray imaging was done at the beginning of the study and on day 10. Imaging was evaluated by two independent blinded radiologists and it indicated that only eight (25%) patients in favipiravir group showed more than 50% improvement in chest X-ray imaging while in lopinavir-ritonavir group only six (20%) patients represented improvement (*P = *0.330) (Figure 2).

Hospitalization lasted for 9 days (IQR, 8-12 days) in the favipiravir group and 12 days (IQR, 10-16 days) in the lopinavir-ritonavir group (*P = *0.030). This result shows that the hospitalization period was significantly lower in the favipiravir group. The mortality results also showed that three (9.37%) patients in the favipiravir group and four (13.33%) patients in the lopinavir-ritonavir group expired during the study (*P = *0.463).

## Discussion

This study evaluated the efficacy and safety of favipiravir in comparison with lopinavir-ritonavir in COVID-19 cases. The main finding of this study brings the fact that favipiravir can alleviate the clinical symptoms more quickly than lopinavir-ritonavir. Also, this agent can shorten the hospitalization period in COVID-19 cases. However, favipiravir was not able to diminish the mortality rate or improve radiological status significantly. The baseline characteristics of patients in both groups were not different statistically, showing the minimal effects of underlying variables. 

Favipiravir is a synthetic prodrug, firstly discovered while assessing the antiviral activity of chemical agents active against the influenza virus in the chemical library of Toyoma chemicals (8). This molecule acts as a substrate for the RNA-dependent RNA-polymerase (RdRp) enzyme, which is mistaken by the enzyme as a purine nucleotide, thus inhibiting its activity and leading to termination of viral protein synthesis (9). On the other hand, the proteases encoded by most viruses play a crucial role in the viral life cycle. The protease inhibitors such as lopinavir-ritonavir competitively bind to the substrate site of the viral protease (10). This enzyme is responsible for the post ­ translational proteolysis of a polyprotein precursor and the release of functional viral proteins, allowing them to function correctly and individually in replication, transcription, and maturation (11). Protease inhibition results in the production of immature virus particles (12). Hence, favipiravir may have a wide antiviral effect in comparison with lopinavir-ritonavir which can explain the current effects of favipiravir in controlling the clinical symptoms.

The shorter hospitalization period in the favipiravir group may be due to better clinical symptoms control. However, the ICU need was not different in both groups. This may be due to the other COVID-19 complications, such as embolism, which is not related to antiviral therapy (13).

Data on radiological changes also was not different in both groups because the follow-up time for these changes was relatively short in this study. In fact, this is far from mind to see significant radiological changes in 10 days (14). Another finding of this study implies that the clinical symptoms recovery is not necessarily associated with radiological changes.

The mortality rate in both groups was similar, and there was not any significant difference in this regard. These findings should be interpreted cautiously as a result of the small sample size in the current study. Favipiravir was not easily available in the Iranian pharmaceutical market and the problem of limited resources to purchase this agent was the main limitation of the study (15). Further trials with larger sample sizes may be required in this regard.

Ivashchenko *et al.* reported the clinical effects of favipiravir in COVID-19 cases. In the pilot stage of phase II/III clinical trial, SARS-CoV-2 viral clearance was shown in 62.5% of patients receiving favipiravir within 4 days. However, viral clearance is not a good indicator to find the clinical benefits of favipiravir. Moreover, the drug was safe and well-tolerated. Our findings on recovery on days 4 and 5 were similar to Ivashchenko *et al.* study (16).

Cai *et al*. reported the results of an open-label study regarding clinical usage of favipiravir in COVID-19. They administered favipiravir at a dose of 1600 mg twice daily on day 1 and 600 mg twice daily from day 2 to 14. Their control group also received lopinavir-ritonavir at a dose of 400/100 mg twice daily from day 1 to 14. They found a shorter viral clearance median time in the favipiravir group. Also, significant improvement in chest CT was reported in the favipiravir group. This finding was not significant in the current study that is maybe due to the longer follow-up period in Cai *et al.* study. Similar to our study, favipiravir administration was associated with lower adverse effects in comparison with lopinavir-ritonavir. The hepatotoxicity caused by lopinavir may be due to its metabolism by the liver, which is largely metabolized by the cytochrome P450 system (CYP3A4), resulting in the production of a toxic intermediate (17). 

Udwadia *et al.* assessed the efficacy and safety of favipiravir in adults with mild to moderate COVID-19. In this study, favipiravir was administered at a dose of 1800 mg twice daily on day 1 and 800  mg twice daily from day 2 to 14. The control group received only standard supportive care. They found that it lasted 3 days (median) to clinical improvement in the favipiravir group and 5 days in the control group. They determined clinical improvement by clinician assessment and defined it as the recovery of fever, respiratory rate of ≤20 breaths/minute, oxygen saturation ≥98% without oxygen supplementation, and cough relief maintained for ≥72 h. Similar results also were seen in the current study (18). 

In conclusion, early administration of oral favipiravir may reduce the duration of clinical signs and symptoms in patients with COVID-19, as well as hospitalization period. The mortality rate also should be investigated in future clinical trials. 

**Table 1:** The baseline characteristics of patients

Characteristic	favipiravir group (n = 32)	lopinavir-ritonavir group (n = 30)	*P*-value
**Median age (IQR)-years**	54.5 (42-64.5)	62 (48-66)	0.200
**Age category - no. (%)**
**<50 years**	15 (46.87)	8 (26.66)	0.413
**50 to <70 years**	11 (34.37)	18 (60)	
**≥70 years**	6 (18.75)	4 (13.33)	
**Male sex - no. (%)**	15 (46.87)	21 (70)	0.067
**Coexisting conditions - no. (%)**	17 (53.12)	13 (43.33)	0.832
**Hypertension**	5 (15.62)	3 (10)	
**Diabetes**	6 (18.75)	7 (23.33)	
**Cardiovascular disease**	6 (18.75)	3 (10)	
**Rheumatoid arthritis**	4 (12.5)	1 (3.33)	
**Hypothyroidism**	2 (6.25)	2 (6.66)	
**Malignancy**	2 (6.25)	1 (3.33)	
**Chronic kidney disease**	0	2 (6.66)	
**Asthma**	0	2 (6.66)	
**Obesity**	1 (3.12)	1 (3.33)	
**Multiple sclerosis**	1 (3.12)	0	
**Dyslipidemia**	0	1 (3.33)	
**Benign prostatic hyperplasia**	0	1 (3.33)	
**Gout**	0	1 (3.33)	
**Time from symptoms onset to intervention (IQR)-days**	7 (7-10)	6 (5-8)	0.357
**Baseline oxygen saturation at admission (%)**	84 ± 6	85 ± 3	0.748
